# Pre-Steady-State and Steady-State Kinetic Analysis of Butyrylcholinesterase-Catalyzed Hydrolysis of Mirabegron, an Arylacylamide Drug

**DOI:** 10.3390/molecules29102356

**Published:** 2024-05-16

**Authors:** Zukhra Shaihutdinova, Patrick Masson

**Affiliations:** Laboratory of Biochemical Neuropharmacology, Kazan Federal University, Kremlevskaya St. 18, 420008 Kazan, Russia; shajhutdinova.z@mail.ru

**Keywords:** arylacylamide, butyrylcholinesterase, burst, drug metabolism, hysteretic enzyme, mirabegron

## Abstract

The β-adrenergic drug Mirabegron, a drug initially used for the treatment of an overactive bladder, has new potential indications and is hydrolyzed by butyrylcholinesterase (BChE). This compound is one of the only arylacylamide substrates to be catabolized by BChE. A steady-state kinetic analysis at 25 °C and pH 7.0 showed that the enzyme behavior is Michaelian with this substrate and displays a long pre-steady-state phase characterized by a burst. The induction time, *τ*, increased with substrate concentration (*τ* ≈ 18 min at maximum velocity). The kinetic behavior was interpreted in terms of hysteretic behavior, resulting from a slow equilibrium between two enzyme active forms, E and E′. The pre-steady-state phase with the highest activity corresponds to action of the E form, and the steady state corresponds to action of the E′ form. The catalytic parameters were determined as *k_cat_* = 7.3 min^−1^ and *K_m_* = 23.5 μM for the initial (burst) form E, and *k_cat_* = 1.6 min^−1^ and *K_m_* = 3.9 μM for the final form E′. Thus, the higher affinity of E′ for Mirabegron triggers the slow enzyme state equilibrium toward a slow steady state. Despite the complexity of the reaction mechanism of Mirabegron with BChE, slow BChE-catalyzed degradation of Mirabegron in blood should have no impact on the pharmacological activities of this drug.

## 1. Introduction

**Scheme 1:**
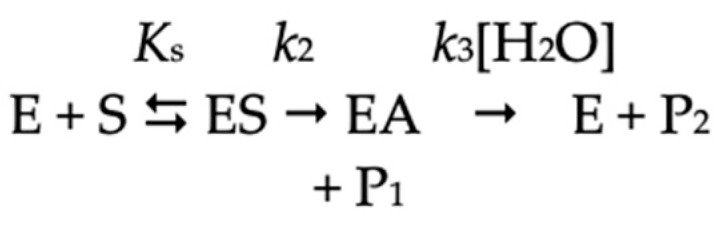
Michaelis–Menten model for hydrolysis of a neutral substrate by BChE.

Butyrylcholinesterase (BChE) [1] is structurally and functionality related to acetylcholinesterase (AChE), the key enzyme of the cholinergic system that terminates the action of the neurotransmitter acetylcholine [2]. In humans, BChE is present in numerous organs and tissues, in particular in plasma. However, the physiological functions of BChE are not clear: the enzyme may play the role of a backup of AChE and a regulator in the cholinergic system; a role in cell differentiation and embryogenesis; and a role in the metabolism of fatty acids and ghrelin, the hunger hormone [1,2,3]. Moreover, BChE, in addition to exhibiting esterase activity, is a promiscuous enzyme that hydrolyzes numerous compounds of toxicological or medical interest [3]. Among them are arylacylamides (AAAs). BChE slowly hydrolyzes these substrates according to the classical two-kinetic-step Michaelis–Menten hydrolysis mechanism (Scheme 1), where ES is the enzyme–substrate complex (*K*_s_ is the dissociation constant of ES) and EA is the acylated enzyme that is subsequently deacylated by the nucleophilic attack of water, acting as a co-substrate. Very fast deacylation takes place with a rate constant *k*_3_. P_1_ and P_2_ are the reaction products.

The mechanistic Scheme 1 is described by the simple Michaelis–Menten rate equation (Equation (1)):

(1) $$v=\frac{k_{\mathrm{cat}}\left[E\right]\left[S\right]}{K_{m}+\left[S\right]}$$

with the Michaelis constant, *K_m_:*

(2) $$K_{m}=\frac{K_{s}k_{3}}{\left(k_{2}+k_{3}\right)}=\frac{K_{s}}{\left(1+\frac{k_{2}}{k_{3}}\right)}$$

and the catalytic constant (turnover), *k_cat_*:

(3) $$k_{\mathrm{cat}}=\frac{V_{max}}{\left[E\right]}=\frac{k_{2}k_{3}}{k_{2}+k_{3}}$$

In Equation (3), *V_max_* is the maximum velocity at the saturating substrate concentration [*S*] and [*E*] is the enzyme molar concentration.

In the case of AAA substrates, where acylation (*k*_2_) is the rate-limiting step (*k*_2_ *<< k*_3_), the catalytic constant (*k_cat_*) is equal to the acylation constant *k*_2_ and *K_m_* = *K_s_*, the dissociation of the enzyme–substrate complex ES [4,5]. Then, the bimolecular rate constant of the reaction, *k_cat_/K_m_*, is:

(4) $$\frac{k_{cat}}{K_{m}}=\frac{k_{2}}{K_{S}}$$

**Figure 1:**
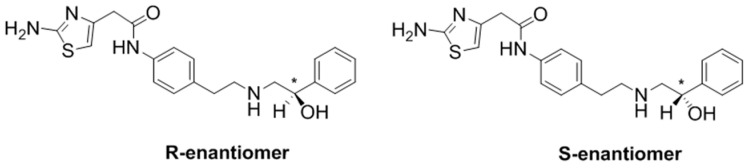
Structure of Mirabegron, R and S stereoisomers.

Only one AAA drug, the substrate of BChE, has been used in human medicine for its pharmacological properties so far [3]. Racemic Mirabegron (CAS number 223673-61-8;2-amino-N-[4-[2-[[(2R)-2-hydroxy-2-phenylethyl]amino]ethyl]phenyl]-4-thiazoleacetamide) (Figure 1), also marketed as MYRBETRIQ, BETANIS and BETMIGA, is a β_3_-adrenergic agonist initially used for the treatment of an overactive bladder (OAB) [6]. However, this drug has several other potential uses, for example, as an anti-obesity drug [7], and—as a consequence of browning adipose tissues—an anticancer agent [8]. Thus, given the renewed interest in this drug, it is important to revisit its metabolism, in particular the mechanism of its hydrolytic inactivation in blood circulation.

It should be noted that the *s*-isomer of the molecule, Mirabegron (S)-isomer (CAS number 1796931-48-0), has also been indicated for the treatment of an OAB, but it is not marketed as an approved pharmaceutical. No work has been reported on the metabolism of this isomer.

Earlier studies on racemic Mirabegron degradation showed that human AChE does not hydrolyze Mirabegron, but that plasma BChE participates in its catabolism. BChEhydrolyzes the amide bond [9,10]. However, except for *K_m_* (=15.2 μM) at 37 °C [9],the kinetic parameters of the reaction were not determined and the mechanism of hydrolysis was not fully investigated. Moreover, the kinetics were not investigated under steady-state conditions, but were investigated by monitoring the reaction product released after fixed incubation times. Yet, the ChE-catalyzed hydrolysis of known AAAs displays interesting characteristics: acylation is the rate-limiting step [4,5] and a “burst” before the establishment of a steady state can be observed [5,11]. Therefore, the present work was aimed at solving unanswered mechanistic questions about the BChE-catalyzed hydrolysis of Mirabegron. 

## 2. Results and Discussion

**Figure 2:**
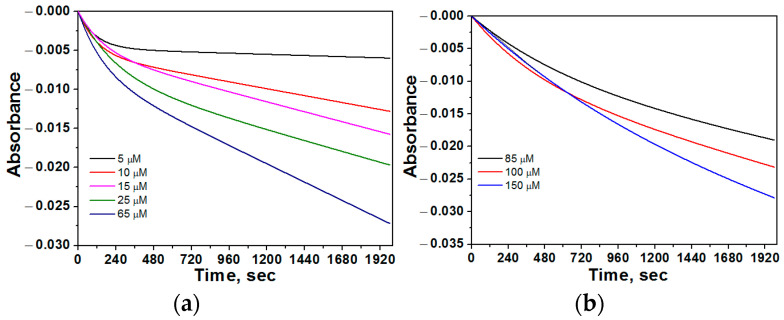
BChE-catalyzed hydrolysis kinetics of Mirabegron in 0.1 M phosphate buffer pH 7.0 at 25 °C: (**a**) from 5 to 65 μM Mirabegron; (**b**) from 85 to 150 μM Mirabegron. The change in absorbance was monitored at 247 nm.

Our first kinetics measurements of BChE-catalyzed hydrolysis of Mirabegron revealed that establishment of the steady state is long, showing non-linear portions of progress curves preceding the steady-state hydrolysis phase (Figure 2a,b). For each concentration of the substrate, the initial velocity (*v_i_*) was faster than the steady-state velocity (*v_ss_*). This is characteristic of “burst” kinetics (*v_i_* > *v_ss_*). The duration of the burst increased with the substrate concentration.

Progress curves were analyzed using the general integrated rate equation (Equation (5)) that describes the release of the monitored reaction product *P*_1_ as a function of time from the mono-exponential pre-steady-state phase to the steady state.

(5) $$\left[P_{1}\right]=v_{ss}t+\frac{\left(v_{i}-v_{ss}\right)\left(1-\exp\left(-k_{obs}t\right)\right)}{k_{obs}}$$

The reciprocal of the first-order rate constant (*k_obs_*) associated with the pre-steady-state phase is the induction time *τ*.

### 2.1. Pre-Steady-State Hydrolysis of Mirabegron

**Figure 3:**
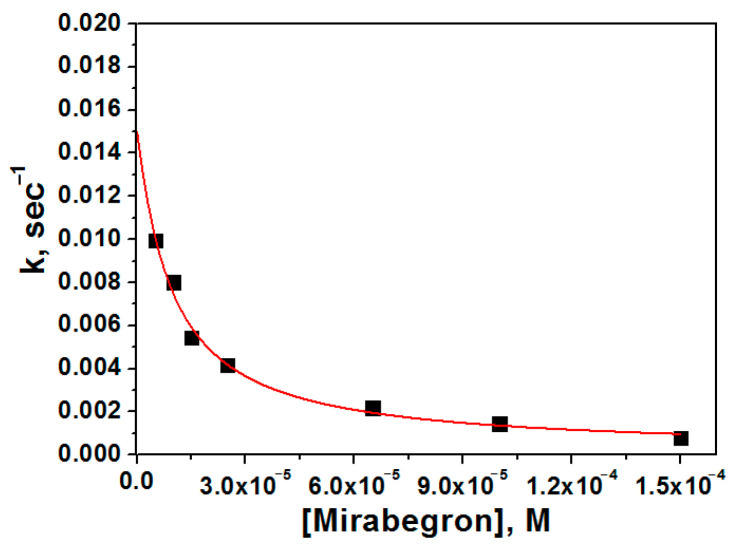
Dependence of the first-order induction rate constant (*k_obs_*) on the Mirabegron concentration. Data fitted to Equation (6) (r^2^ = 0.99).

The first-order rate constant, *k_obs_*, was determined for each substrate concentration. The change in *k_obs_* as a function of [*S*] was monophasic, giving a descending hyperbole (Figure 3). The downward-curved hyperbolic dependence of *k_obs_* as a function of [*S*] has been associated with the existence of a slow equilibrium between enzyme “conformers”, preceding ligand or substrate binding in enzyme mechanisms. Such a kinetic behavior with substrates, coined as “hysteresis”, was analyzed by Frieden [12] and other investigators [13,14]. It results from the existence of two enzyme forms, E and E′, in slow equilibrium, binding of the substrate and a shift in the equilibrium in one or the other direction. The structural differences between E and E′ are always subtle, e.g., a cis/trans isomerization of a key proline residue [14]. In the case of BChE, a molecular modeling study (QM/MM) showed that the enzyme hysteretic behavior depends on a flip of the His438 ring, the catalytic histidine, and part of the catalytic triad Ser198/His438/Glu332 [15]. Thus, the orientation of the histidine ring within the catalytic triad determines the efficiency of proton transfer during the catalytic cycle.

Several mechanistic models can be derived. Hysteretic behavior was already observed for BChE with certain ester substrates [16] and an arylacylamide [17]. A review summarized the results obtained with this enzyme and described the different possible models [11].

**Scheme 2:**
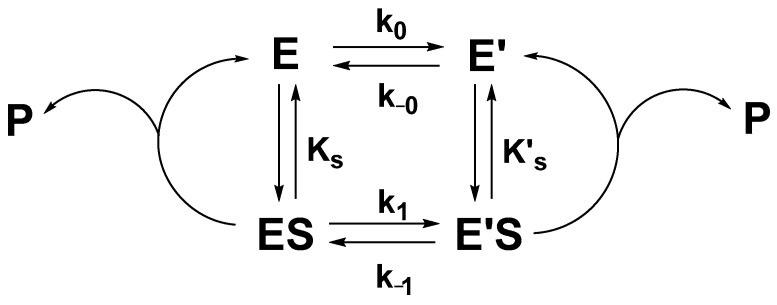
General Frieden model [12] for hysteretic enzymes. *k*_0_ and *k*_−0_, and *k*_1_ and *k*_−1_ are the kinetic constants of the slow equilibria E⇆E′ and ES⇆E′S, respectively. With *K_s_* < *K*′*_s_*, the affinity of the E form for S is higher than the affinity of the E′ form, and then progress curves show an initial burst.

The fact that progress curves show a burst (Figure 2) indicates that both enzyme forms are active, with *v_i_* > *v_ss_*. Thus, the general model (Scheme 2) proposed by Frieden [12] for kinetic analysis of the hysteretic behavior of enzymes can be used to describe the behavior of BChE with Mirabegron as the substrate. 

According to this model, the two active forms of the enzyme, E and E′, in slow equilibrium differ in their binding properties (*K_s_* and *K*′*_s_*, the dissociation constant of ES and ES′ complexes) and catalytic activity (*k_cat_* and *k*′*_cat_* of both forms). Then, the dependence of *k_obs_* on [*S*] can be described by the Frieden general equation (Equation (6)) corresponding to the model in Scheme 2:

(6) $$k_{obs}=\left(\frac{k_{0}+\frac{k_{1}\left[S\right]}{K_{s}}}{1+\frac{\left[S\right]}{K_{s}}}\right)+\left(\frac{k_{-0}+\frac{k_{-1}\left[S\right]}{K_{s}^{\prime }}}{1+\frac{\left[S\right]}{K_{s}^{\prime }}}\right)$$

in which at [*S*] =0, *k_obs_*_,0_ = *k*_0_ + *k*_−0_ and *k_lim_* = *k*_1_ + *k*_−1_ at saturation [*S*]. The dissociation constants *K_s_* and $K_{s}^{\prime }$ are regarded as *K_m_* and $K_{m}^{\prime }$. Frieden’s assumption is perfectly valid in the case of the BChE-catalyzed hydrolysis of an arylacylamide substrate where *k_cat_*= *k*_2_ (cf. Equation (4)). The catalytic parameters of Equation (6) were determined (after rewriting this equation, see Supplementary Information) by non-linear computer fitting of this equation. 

This provided limit values of *k_obs_* = 0.0149 min^−1^ at [*S*] = 0 and *k_lim_* = 0.000146 min^−1^ at infinite time [*S*]. These values fit with the negative hyperbolic dependence of *k_obs_* on [*S*], and thus fit with the fact that both forms, E and E′, are active. Then, this determines a long induction phase that precedes establishment of the steadystate, with 1/*k_obs_* = *τ* ≈18 min, the maximum induction time, at *V_max_*. The sum of *K_s_* + *K′_s_* = 16.5 μM is of the same order as the sum of *K_m_* values (=27.4 μM, cf. Table 1). This indicates that both complexes, ES and E′S, are productive. This is in accordance with the mechanistic model in Scheme 2.

### 2.2. Initial and Steady-State Hydrolysis of Mirabegron

**Figure 4:**
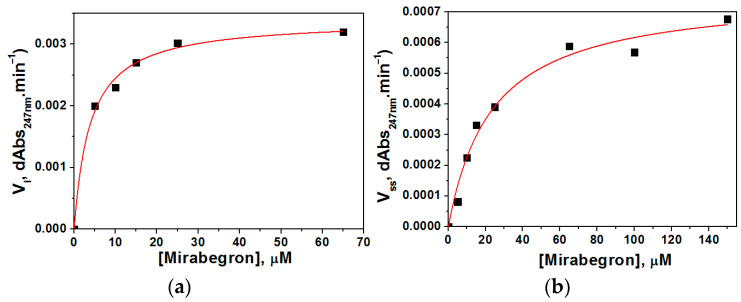
Dependence of initial (*v_i_*) and steady-state (*v_ss_*) rates on the Mirabegron concentration in 0.1 M phosphate, pH 7.0, at 25 °C: (**a**) form E; (**b**) form E′. Data fitted to Equation (1): (**a**) r^2^ = 0.99; (**b**) r^2^ = 0.98.

Analysis of hydrolysis rates as a function of the Mirabegron concentration, initial rates (*v_i_*) and steady-state rates (*v_ss_*) up to 150 μM of Mirabegron showed that the hydrolysis is Michaelian for both enzyme forms (Figure 4a,b). Initial and steady-state catalytic parameters, *K_m_* and *k_cat_*, were determined from non-linear curve fitting to Equation (1) (Figure 4).

The catalytic parameters for both phases, corresponding to the hydrolysis of the substrate by E and E′, are reported in Table 1. As predicted from the existence of a pre-steady state burst and the kinetic model in Scheme 2, *K_m_*_,E_ < *K_m,_*_E′_ and *k*_*cat*,E′_ > *k*_*cat,*E_. The *K_m_* value at 25 °C for the form E′ fits with the value previously reported by Takusagawa [9] of 15.2 μM at 37 °C. The turnover numbers (*k_cat_*) are low, but on the same order as reported *k_cat_* values of BChE for hydrolysis of neutral AAA substrates [5].

At this point, it is important to remember that the energy needed to break an amide bond is much higher than for breakage of an ester bond. Then, because for ChE-catalyzed hydrolysis of ester substrates, acylation (*k*_2_) and diacylation (*k*_3_) are partly rate-limiting (*k*_2_ ≈ *k*_3_) [4,18], it follows that in the case of AAA substrates, acylation is rate-limiting (*k*_2_ << *k*_3_). Therefore, with Mirabegron, as for BChE-catalyzed hydrolysis of other AAAs, acylation is the rate-limiting step (*k*_2_ << *k*_3_) [5]. Then, because *k_cat_*/*K_m_* = *k*_2_/*K*_s_ (Equation (4)), it follows that *K_m_* = *K_s_,* and as for other substrates of BChE, it can reasonably be postulated that the deacylation rate, *k*_3_, is higher than 10,000 min^−1^ (*k*_3_ >> *k*_2_). 

In summary, the high affinity (low *K_m_*) of BChE for Mirabegron and the low catalytic activity (*k_cat_*) of the enzyme provide a bimolecular rate constant (*k_cat_*/*K_m_*) close to 10^6^ M^−1^ min^−1^ for the E form and higher than 10^4^ M^−1^ min^−1^ for the E′ form. Thus, taking into account the standard posology of this drug (50 mg/day in one tablet), the maximum concentration of Mirabegron in blood is always very low, much lower than reported *K_m_* values, and hydrolysis takes place under first-order conditions ([*S*] << *K_m_*). The average plasma concentration of BChE ([*E*]) in human blood (5 mg/L, i.e.,1.47 × 10^−8^ M) then allows for hydrolysis of Mirabegron with a first-order rate constant (*k*_cat_/*K*_m_)·[*E*] is less than 0.026 min^−1^ and less than 0.0014 min^−1^ for the E form and E′ form, respectively. Therefore, under such low metabolic rates, BChE-catalyzed degradation of Mirabegron does not impair the β-adrenergic action of this drug.

## 3. Materials and Methods

### 3.1. Chemicals and Enzymes

Racemic Mirabegron was purchased from Sigma-Aldrich (Saint Louis, MO, USA). A stock solution (50 mM) of Mirabegron was made with DMSO. Butyrylthiocholine iodide (BTC) and dithio-bisnitrobenzoic acid (DTNB) were also from Sigma-Aldrich (Saint Louis, MO, USA). Stock solutions of BTC (0.1 M) were made with water and stored at −20 °C. A stock solution of DTNB (10 mM) was prepared with 0.1 M phosphate buffer, pH 7.0, supplemented with 15 mg/10 mL sodium hydrogeno-carbonate. This solution was stored at +4 °C and is light-sensitive. Echothiophate iodide was a gift from Biobasal AG (Basel, Switzerland). A stock solution of 0.1 M echothiophate was prepared with water and stored at −20 °C.

Human BChE was highly purified to homogeneity from human plasma Cohn fraction IV-4 [19] and was a gift from Dr. O. Lockridge (UNMC, Omaha, NE, USA). The enzyme is a homo-tetramer of 340 kDa. The preparation was diluted in 0.1 M sodium phosphate buffer, pH 7.0, to an activity of 45 international units/mL with 1 mM BTC as the substrate at 25 °C (one international unit (I.U) corresponds to the number of micromoles of BTC hydrolyzed per minute).

### 3.2. Enzyme Titration

The diluted BChE solution was titrated according to the sampling method of Leuzinger [20] using echothiophate iodide as the titrant. During the titration processes, the activity of the enzyme was checked, using the reference method of Ellman [21] with 1 mM BTC in 0.1 M phosphate buffer, pH 7.0, in the presence of DTNB (0.01 M) as the thiocholine-reacting chromogenic dye. The temperature was 25 °C. A titration plot was built (see Supplementary Information) and led to an active site concentration in the diluted 45 I.U/mL BChE solution of 1.02 × 10^−6^ M.

### 3.3. Steady-State Hydrolysis of Mirabegron

**Figure 5:**
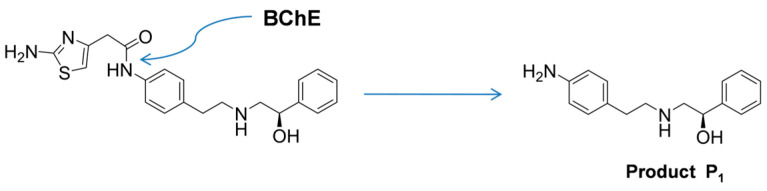
Hydrolysis scheme of Mirabegron. BChE hydrolyzes the arylamide bond.

The BChE-catalyzed hydrolysis of Mirabegron was studied in 0.1 M phosphate buffer, pH 7.0, at 25 °C. The Mirabegron concentration ranged from 5 to 150 μM. The final concentration of DMSO was 1% in assays. At this concentration, DMSO has only a mild effect on enzymes [22,23]. This concentration does not affect either the hydrolytic mechanism or the catalytic activity of the enzyme. We also tested methanol at a 5% final concentration as the co-solvent. However, methanol acts as a nucleophilic competitor of water [24] and might have altered the catalytic mechanism. Thus, we did not use this methanol. The enzyme concentration in assays was 1.02 × 10^−7^ M. Hydrolysis was spectrophotometrically monitored at 247 nm by following the decrease in absorbance (release of (R)-2((4-aminophenethyl) amino)-1-phenylethanol, the amine product P_1_) (Figure 5 and Figure S1). The difference in the absorptivity constant at 247 nm (∆ε) between Mirabegron and product P_1_ was 9100 ± 300 M^−1^cm^−1^ (see Supplementary Information). These kinetic measurements were performed on a temperature-controlled double beam spectrophotometer (model TUV9DCS, SILab China, LabKontsept, Saint Petersburg, Russia).

Experiments were performed in triplicate. First-order rate constants and catalytic parameters for pre-steady-state and steady-state phases were determined from non-linear curve fitting of kinetic data using the software OriginPro 8.5 (Originlab Co., Northampton, MA, USA).

## 4. Conclusions

This kinetic study devoted to a β-adrenergic drug, Mirabegron, catabolized by BChE provides the missing information that may have not been obtained in initial kinetics studies for technical reasons [9]. The results showed that BChE-catalyzed hydrolysis of Mirabegron displays a long pre-steady-state phase characterized by a burst of 18 min at *V_max_*. The pre-steady-state phase was interpreted in terms of enzyme hysteretic behavior according to the general model of Frieden, i.e., the existence of two active enzyme forms in slow equilibrium [12]. From a pharmacological point of view, both the complex mechanism of BChE and the slow BChE-catalyzed degradation of Mirabegron in blood must have no impact on the pharmacological activity of this drug.

The results obtained in the present study also provide new evidence about the complexity of cholinesterase-catalyzed reactions. Although the existence of long induction phases preceding the establishment of steady-state kinetics of ChEs has long been known, systematic kinetic and molecular modeling investigations of this catalytic behavior with certain substrates need more attention. Yet, the existence of multiple enzyme conformers has long been recognized and their connection with slow-binding inhibition/reactions of type C has been proposed [25]. 

Although little is known about the molecular mechanism of cholinesterase hysteretic catalytic behavior, molecular modeling using a QM/MM approach suggested that it is controlled by a flip of the catalytic triad histidine ring [15],as we have also found. This flip tunes the catalytic activity of the enzyme. Because of cross talk between substrate binding sites and the catalytic center, the flip depends on the nature of substrate, mutant enzyme, and medium composition. However, very few structural and molecular dynamic data are available to date, and we do not know whether this histidine flip is the sole mechanism that accounts for hysteresis of cholinesterases, where transient phases are lags of bursts. In particular, in the case of Mirabegron and another arylamide substrate, ATMA [5,11], the pre-steady-state phase is a burst, while for ester substrates, lag phases are observed [11,16,17]. This difference in pre-steady-state behavior is important, owing to the catalytic mechanism particularity of arylacylamide substrate hydrolysis (*k*_2_ << *k*_3_), while for ester substrates, acylation and deacylation are partly rate-limited (*k*_2_ ≈ *k*_3_). Therefore, further works, including an in silico approach (QM/MM) to BChE-catalyzed hydrolysis of Mirabegron as a model arylacylamide substrate, are needed. Moreover, the physiological function(s) of BChE is(are) not perfectly known [2]. It is obvious that the neurotransmitter acetylcholine is one of the physiological substrates, but we cannot rule out that an endogenous arylacylamide is also a physiological substrate. Thus, further works with Mirabegron could shed light on the alternative functions of BChE, involving its promiscuous arylacylamidase activity.

Also, we must point out that the possible functional significance of cholinesterase’s hysteretic behavior is not known; it can provide slower (lag times) or faster (burst) responses upon binding of certain substrates/ligands compared to classical fast responses upon binding/reaction. This catalytic behavior may have important physiological, pharmacological and toxicological consequences, e.g., damping the response of targeted regulatory enzymes and enzymes located in micro-compartments where re-binding of the ligand/substrate may take place after dissociation of complexes. A recent work pointed out the importance of hysteretic (also called “allokairy”) regulation of a promiscuous monomeric esterase [26]. Thus, the tight regulation of biological systems implies the fine tuning of enzyme activity for optimizing physiological responses. Allosteric regulation involves spatial cooperativity between protein subunits, while hysteretic regulation results from temporal cooperativity between different protein conformational states. While allostery involves multiple cooperative bindings, in hysteresis (allokairy), the modulation of the activity of monomeric and oligomeric enzymes only depends on the nature of substrates and/or the presence of modulators. Such modulators can be either small molecules or other proteins/macromolecules. In this respect, identification of endogenous modulators is important for understanding the pathological consequences of metabolic dysregulations. Therefore, knowledge of the molecular basis of slow conformational selection versus a slow induced fit in ChE substrates and ligand binding deserves particular attention owing to the physiological, pharmacological and toxicological importance of these enzymes. Such regulatory enzyme catalytic behaviors may be of importance when their substrates are pleiotropic drugs.

## Figures and Tables

**Table 1:** Catalytic parameters (±SE) for BChE-catalyzed hydrolysis of Mirabegon in phosphate buffer, pH 7.0 at 25 °C.

	BChE form E	BChE form E′
*K* *_m_*	3.9 ± 0.5 μM	23.5 ± 3.9 μM
*k_cat_*	7.32 ± 0.21 min^−1^	1.63 ± 0.08 min^−1^
*k_cat_*/*K_m_*	1.8 × 10^6^ ± 0.28 × 10^6^ M^−1^min^−1^	7.1 × 10^4^ ± 1.4 × 10^4^ M^−1^min^−1^

## Data Availability

The data presented in this study are available in article and Supplementary Materials.

## Supplementary Materials

The following supporting information can be downloaded at: https://www.mdpi.com/article/10.3390/molecules29102356/s1, Figure S1: Calibrations of Mirabegron; Figure S2: Calibrations of Mirabegron metabolite MI 16 (hydrolysis product 1); Figure S3: Titration plot of BChE with echotiophate; Equations (S1): Determination of parameters of the Frieden equation.

## Abbreviations

AAA, arylacylamide; AChE, acetylcholinesterase; BChE, butyrylcholinesterase; ChE, cholinesterase.
