# Supplementary material for: Pre-Steady-State and Steady-State Kinetic Analysis of Butyrylcholinesterase-Catalyzed Hydrolysis of Mirabegron, an Arylacylamide Drug
Source: Molecules. 2024 May 16;29(10):2356. doi: 10.3390/molecules29102356 (PMC11124411; doi:10.3390/molecules29102356)
Supplement: Supplementary file 1 [file molecules-29-02356-s001.zip › molecules-2998073-supplementary.pdf]

Supplementary Materials

Pre-Steady-State and Steady-State Kinetic Analysis of Butyrylcholinesterase-Catalyzed Hydrolysis of Mirabegron, an Arylacylamide Drug

Zukhra Shaihutdinova and Patrick Masson *

Laboratory of Biochemical Neuropharmacology, Kazan Federal University, Kremlevskaya St. 18,
420008 Kazan, Russia; shajhutdinova.z@mail.ru
* Correspondence: pym.masson@free.fr

Figure S1: Calibrations of Mirabegron

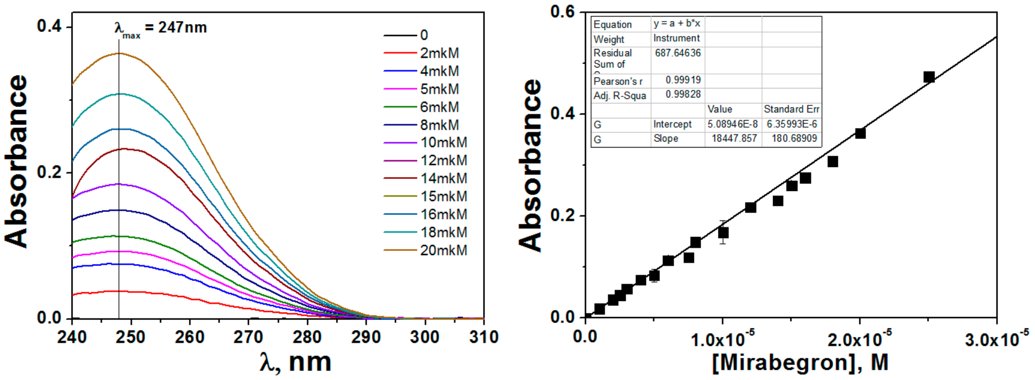

Extinction coefficient of Mirabegron ε = 18447.8±180.6 $M^{-1}cm^{-1}$

Figure S2. Calibrations of Mirabegron metabolite MI 16 (hydrolysis product 1)

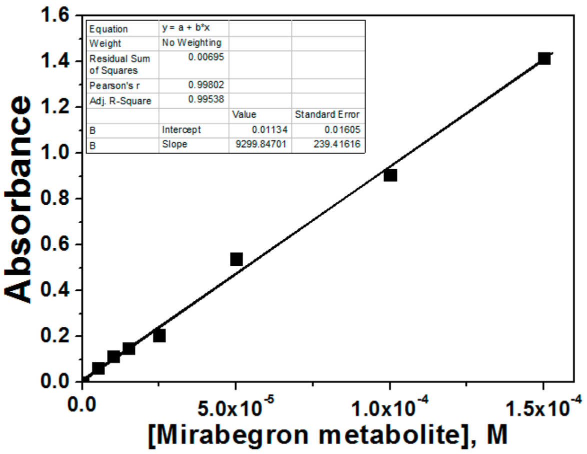

Extinction coefficient of mirabegron metabolite MI 16 (Product 1) ε = 9299.8±239.4 $M^{-1}cm^{-1}$

The difference in the absorptivity constant at 247 nm (Δε) between mirabegron and product P_1 is 9148.01±325.03 $M^{-1}cm^{-1}$

Figure S3. Titration plot of BChE with echotiophate

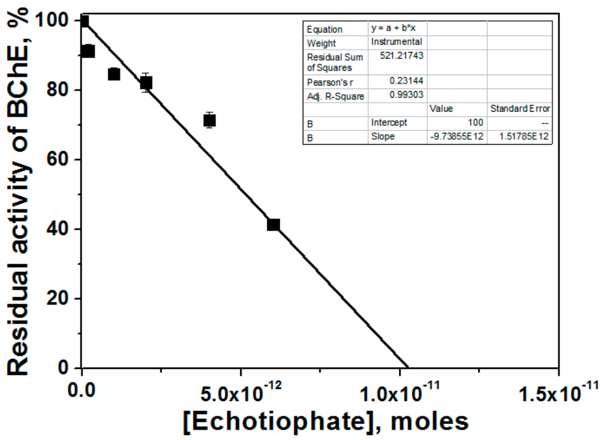

Equations S1. Determination of parameters of the Frieden equation

Equation 2 in the text can be rewritten as:

$$k_{obs} = \frac{(k_0 + k_{-0}) + \left(\frac{k_0 + k_{-1}}{K_s'} + \frac{k_1 + k_{-0}}{K_s}\right)[S] + \left(\frac{k_1 + k_{-1}}{K_s K_s'}\right)[S]^2}{1 + \left(\frac{1}{K_s} + \frac{1}{K_s'}\right)[S] + \left(\frac{1}{K_s K_s'}\right)[S]^2} = \frac{a + bx + c^2}{1 + dx + ex^2}$$

with

$$a = k_0 + k_{-0}$$

$$\frac{c}{e} = k_1 + k_{-1}$$

$$\frac{d}{e} = K_s + K_s'$$

$$b = \left(\frac{k_0 + k_{-1}}{K_s'} + \frac{k_1 + k_{-0}}{K_s}\right)$$

$$c = \left(\frac{k_1 + k_{-1}}{K_s K_s'}\right)$$

$$d = \left(\frac{1}{K_s} + \frac{1}{K_s'}\right)$$

$$e = \left(\frac{1}{K_s K_s'}\right)$$
